# The vertical hip fracture – a treatment challenge. A cohort study with an up to 9 year follow-up of 137 consecutive hips treated with sliding hip screw and antirotation screw

**DOI:** 10.1186/1471-2474-13-171

**Published:** 2012-09-13

**Authors:** Anders Enocson, Lasse J Lapidus

**Affiliations:** 1 Department of Clinical Science and Education, Orthopaedic Unit, Karolinska Institutet, Stockholm Söder Hospital, 118 83, Stockholm, Sweden

**Keywords:** Hip fracture, Osteosynthesis, Basicervical fracture, Vertical hip fracture

## Abstract

**Background:**

Femoral neck fractures with a vertical orientation have been associated with an increased risk for failure as they are both axial and rotational unstable and experience increased shear forces compared to the conventional and more horizontally oriented femoral neck fractures. The purpose of this study was to analyse outcome and risk factors for reoperation of these uncommon fractures.

**Methods:**

A cohort study with a consecutive series of 137 hips suffering from a vertical hip fracture, treated with one method: a sliding hips screw with plate and an antirotation screw. Median follow-up time was 4.8 years. Reoperation data was validated against the National Board of Health and Welfare’s national registry using the unique Swedish personal identification number.

**Results:**

The total reoperation rate was 18%. After multivariable Logistic regression analysis adjusting for possible confounding factors there was an increased risk for reoperation for displaced fractures (22%) compared to undisplaced fractures (3%), and for fractures with poor implant position (38%) compared to fractures with adequate implant position (15%).

**Conclusions:**

The reoperation rate was high, and special attention should be given to achieve an appropriate position of the implant.

## Background

Femoral neck fractures with a vertical orientation are fractures with special characteristics and whose optimal treatment remains controversial. They are both axial and rotational unstable and experience increased shear forces compared to the conventional and more horizontally oriented femoral neck fractures [1]. The Pauwels classification, originally published in 1935 is widely used to classify femoral neck fractures according to the orientation of the fracture line. A Pauwels type 3 is a vertically oriented fracture with >50˚ inclination angle from the horizontal line on an anterio-posterior (AP) radiograph [1]. The basicervical fracture is a vertically oriented fracture at the junction between the intertrochanteric region and the femoral neck [2] and comprises only 1.8% of all hip fractures [3]. The treatment options for these fractures include a primary hip arthroplasty, internal fixation using various implants such as two or more cannulated screws, fixed angle devices; sliding hip screw (SHS) or intramedullary nail, or the combination of a SHS and an additional screw to gain rotational stability. As it can be difficult to separate basicervical and Pauwels type 3 fractures from each other, especially before reduction of displaced fractures [4], and they are biomechanically closely related we suggest they can be analysed together. We have used the combination of a SHS and an additional anti rotation screw (ARS) for these fractures since long, and in this study we present our results from a consecutive series of 137 patients followed for up to 9 years. The primary aim of the study was to analyse risk factors for reoperation of these fractures.

## Methods

**Figure 1:**
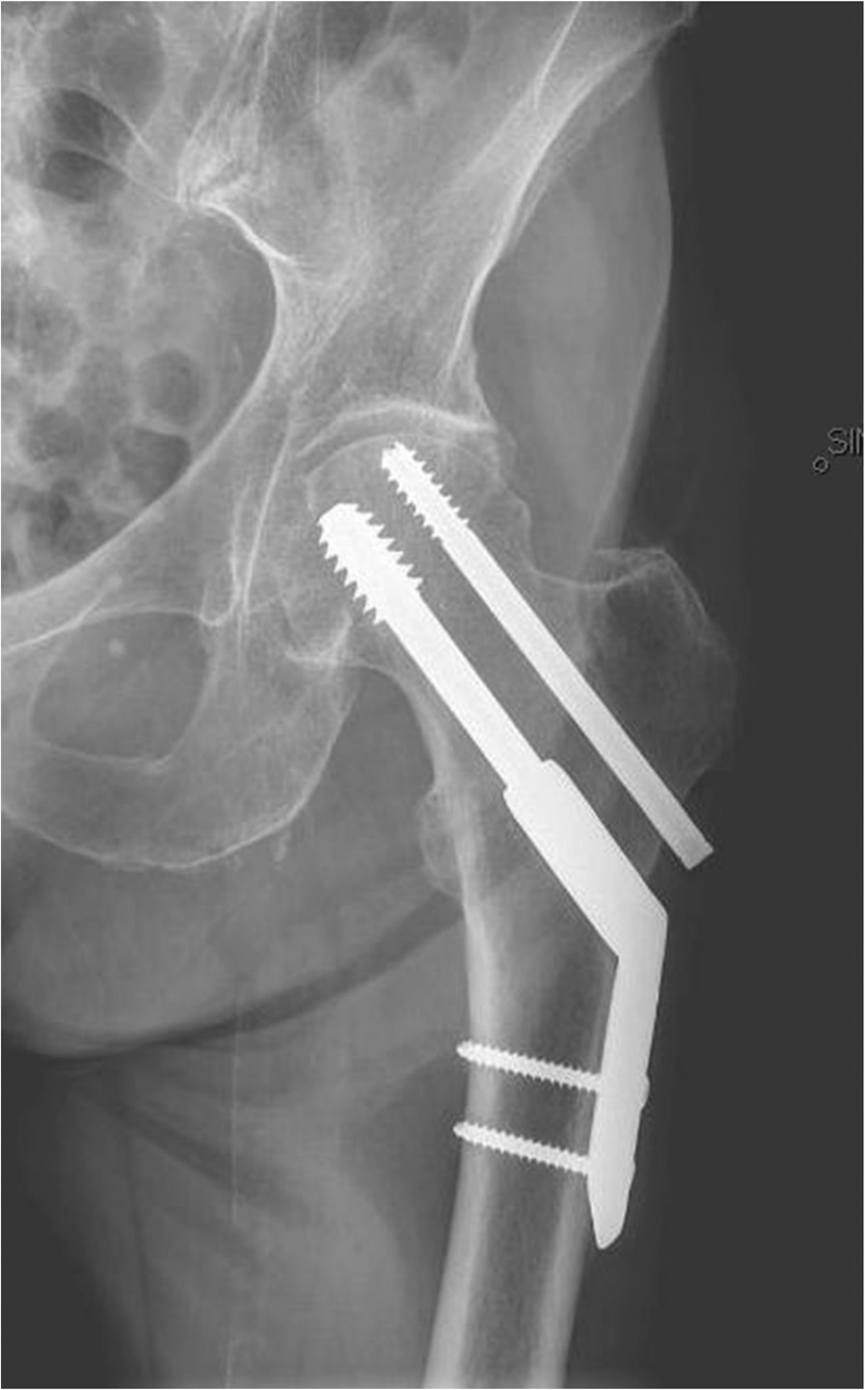
A sliding hip screw (SHS) with a 2-hole plate plus an antirotation screw (ARS) in a patient with a basicervical fracture

The study was conducted at the Department of Orthopaedics at Stockholm Söder Hospital between January 1, 2003, and December 31, 2009. During the study period all consecutive patients; a series of 137 hips in 136 patients (77 females) with a non-pathological hip fracture classified as basicervical (n = 93) or Pauwels type 3 (n = 44), who were operated upon using the combination of a SHS and a superior parallel ARS (Figure 1) were identified and included in the study. Information such as cognitive function, surgical details and others were obtained from the patient records. Patient baseline data are shown in Table 1. All individual patient records were searched until December 31, 2011, or death, in order to find information about all reoperations. In addition, the unique Swedish personal identification number was used to perform a search in the National Board of Health and Welfare’s national registry to find patients who had been treated elsewhere in Sweden for a reoperation up to December 31, 2011. Two such cases were found and their data were included in the analysis. The median follow-up time was 2.3 years (0-8.8) for all cases and 4.8 years (2.2-8.8) for those still alive on December 31, 2011.

**Table 1:** Baseline data for all patients included (n = 137)

**Age median (range)**	82.9 (29–98)
**Gender n (%)**	
Female	77 (56)
Male	60 (44)
**Cognitive dysfunction n (%)**	
No	111 (81)
Yes	26 (19)
**ASA class n (%)**	
1**–**2	42 (31)
3-4	95 (69)
**Primary fracture type n (%)**	
Basicervical	93 (68)
Pauwels type 3	44 (32)
**Implant n (%)**	
2-hole plate SHS and ARS	120 (88)
4-hole plate SHS and ARS	17 (12)

*SHS* = sliding hip screw.

*ARS* = anti rotation screw.

### Surgical procedure

Spinal anaesthesia was the standard anaesthesiological procedure. All patients were operated upon using a hip traction table where fluoroscopy guided closed reduction was performed prior to internal fixation. The SHS used in the study was a dynamic hip screw (DHS) with a 2-hole or a 4-hole plate (Synthes, West Chester, PA, USA) and the ARS used was a cannulated 7.3 mm Olmed screw (DePuy/Johnson & Johnson, Warsaw, IN, USA). The total numbers of surgeons were 57. In the postoperative course patients were allowed weight bearing as tolerated using crutches and mobilised the day after surgery. Patients were given intravenous Flucloxacillin as antibiotic prophylactics and low molecular-weight heparin as thromboembolic prophylactics.

### Radiological analysis

The patient’s pre- and postoperative radiographs were analysed to classify the fracture (undisplaced or displaced) and the postoperative fracture reduction (adequate or poor) and implant position (adequate or poor). Fracture reduction was classified as adequate if the femoral neck angle was <10° varus or <15° valgus compared to the contralateral hip on an AP pelvis radiograph, and the displacement between fracture fragments on AP and lateral radiographs were <3 mm [5]. Implant position was classified as adequate if the SHS on the AP and lateral view respectively was placed; central and central, inferior and central or inferior and posterior in the femoral head, and the distance between the tip of the screw and the bone/cartilage interface distance was <20 mm, and the SHS and the ARS were parallel (<5°), on AP and lateral radiographs [5]. All radiographs were analysed by one of the authors (LJL).

### Statistical analysis

Scale variables were tested using the Mann–Whitney *U*-test for independent groups, and nominal variables were tested by the Fisher’s exact test. All tests were 2-sided. In addition, we used Logistic regression to evaluate factors associated with risk for reoperation. Age, gender, cognitive function, fracture type, fracture displacement, fracture reduction and implant position were tested as independent variables in the model. Firstly, crude associations for each variable were tested in univariable models. Secondly, a multivariable model with all independent variables was used to study the adjusted associations. The associations are presented as odds ratios (ORs) with 95% confidence intervals (CIs). The results were considered significant at p < 0.05. The statistical software used was IBM SPSS Statistics, Version 20 for Windows (SPSS Inc., Chicago, Illinois).

The study was approved by the Regional Ethics Committee in Stockholm June 15, 2011 (reference no. 2011/836-31/3).

## Results

A reoperation was performed in 24 of the 137 patients, giving a total reoperation rate of 18%. There was an increased risk for reoperation for displaced fractures (23/104; 22%) when compared to undisplaced fractures (1/33; 3.0%) (p = 0.009). A poor position of the implant was also associated with an increased risk for reoperation (6/16; 38%) compared to an adequate implant position (18/120; 15%) (p = 0.04).

**Figure 2:**
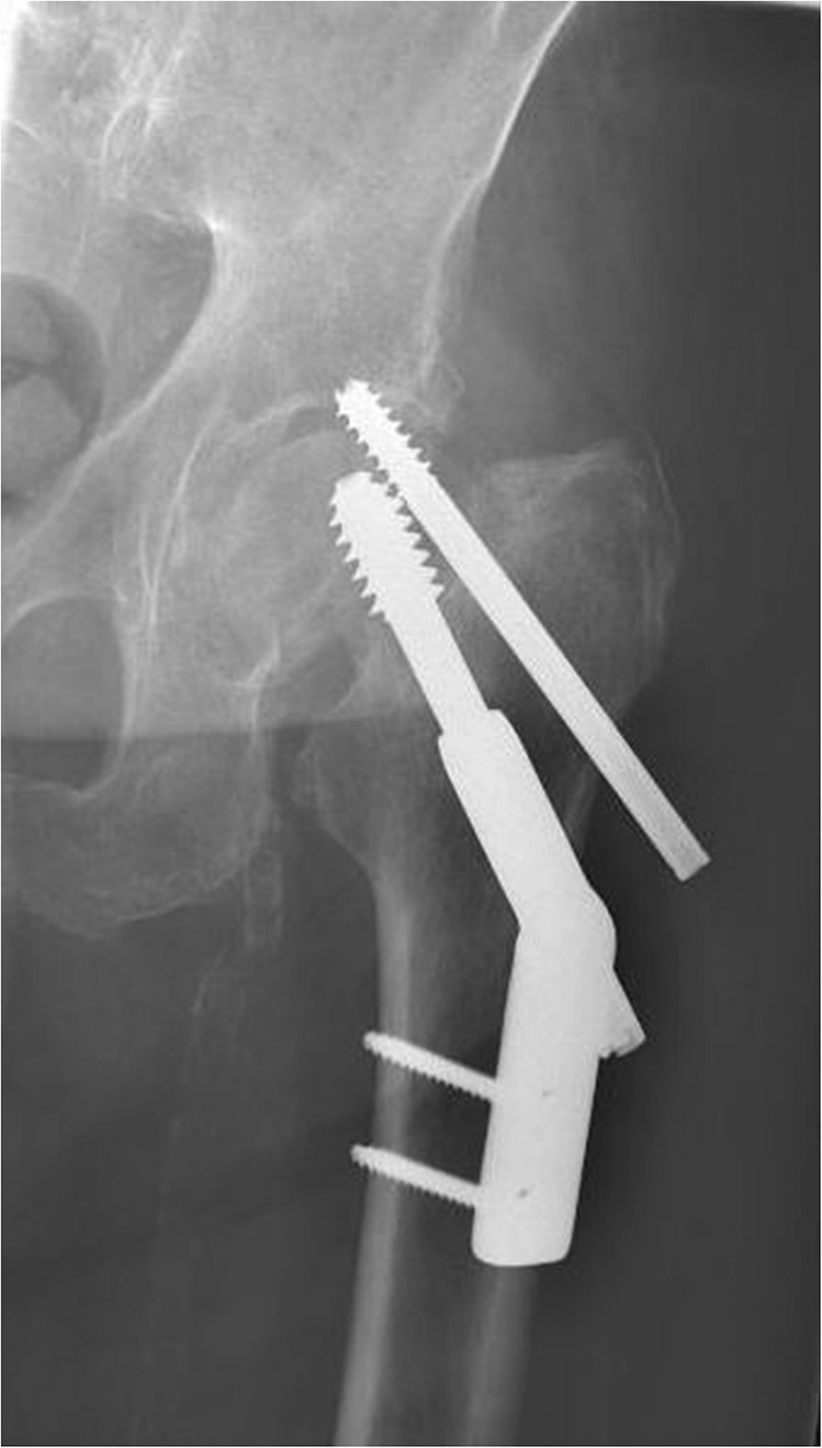
A patient suffering from a cut out of the antirotation screw (ARS) due to a non-union of the fracture

The most common reason for reoperation was a cut out of the SHS or the ARS (n = 6) (Figure 2) followed by lateral pain from the SHS plate or laterally protruding screws (n = 5), a fracture non-union (n = 4), a penetration of the ARS to the joint (n = 3), an avascular necrosis of the femoral head (n = 2), a deep infection (n = 2), a subtrochanteric fracture (n = 1) or a post traumatic osteoarthritis (n = 1) (Table 2).

**Table 2:** Patients with reoperations (n = 24)

**Patient**	**Age**	**Gender**	**Fracture type**	**Primary implant**	**Indication for reoperation**	**Reoperation**	**Time to reoperation**	**History**
**1**	87	Female	Pauwels 3	2 SHS + ARS	Cut out	Girdlestone	11 months	No further operations
**2**	86	Male	Basicervical	2 SHS + ARS	Cut out	Bipolar HA	2 weeks	Deep infection of HA
**3**	87	Female	Basicervical	2 SHS + ARS	Cut out	Girdlestone	1 month	No further operations
**4**	88	Female	Pauwels 3	2 SHS + ARS	Cut out	Unipolar HA	6 months	No further operations
**5**	86	Female	Pauwels 3	2 SHS + ARS	Cut out	THA	3.5 months	No further operations
**6**	79	Female	Basicervical	2 SHS + ARS	Cut out	THA	15 months	No further operations
**7**	43	Female	Basicervical	2 SHS + ARS	Lateral pain	Extraction all	1.5 years	No further operations
**8**	67	Female	Pauwels 3	2 SHS + ARS	Lateral pain	Extraction ARS	3 years	No further operations
**9**	36	Male	Pauwels 3	2 SHS + ARS	Lateral pain	Extraction all	3 years	No further operations
**10**	70	Female	Basicervical	2 SHS + ARS	Lateral pain	Exchange to shorter screws	4 months	Reoperation with IM nail due to trochanteric fracture 19 months after exchange
**11**	55	Female	Pauwels 3	2 SHS + ARS	Lateral pain	Extraction all	2 years	THA due to avascular necrosis 8 months after extraction
**12**	93	Male	Basicervical	2 SHS + ARS	Non-union	Unipolar HA	7 months	No further operations
**13**	63	Male	Pauwels 3	2 SHS + ARS	Non-union	THA	10 months	Plate fixation of periprosthetic fracture of THA after 4 years
**14**	68	Male	Pauwels 3	2 SHS + ARS	Non-union	Bipolar HA	1 month	Revision to THA due to a periprosthetic fracture of HA after 6 years
**15**	61	Female	Basicervical	2 SHS + ARS	Non-union	THA	4 months	No further operations
**16**	73	Male	Basicervical	2 SHS + ARS	Penetration of ARS	Extr of ARS	2 days	Girdlestone performed 8 months later due to non-union
**17**	75	Female	Basicervical	2 SHS + ARS	Penetration of ARS	Exchange to shorter ARS	4 days	No further operations
**18**	85	Male	Basicervical	2 SHS + ARS	Penetration of ARS	Exchange to shorter ARS	5 days	No further operations
**19**	93	Female	Basicervical	2 SHS + ARS	Deep infection	Extraction all	1 year	No further operations
**20**	70	Male	Basicervical	2 SHS + ARS	Deep infection	Girdlestone	2 years	No further operations
**21**	65	Male	Pauwels 3	2 SHS + ARS	Avascular necrosis	THA	1.5 years	Deep infection after THA
**22**	90	Female	Basicervical	4 SHS + ARS	Avascular necrosis	THA	10 months	No further operations
**23**	59	Male	Basicervical	2 SHS + ARS	Subtrochanteric fracture	IM nail	24 days	No further operations
**24**	81	Male	Basicervical	2 SHS + ARS	Post traumatic OA	THA	6 months	No further operations

*2 SHS* = 2-hole plate sliding hip screw.

*4 SHS* = 4-hole plate sliding hip screw.

*ARS* = anti rotation screw.

*OA* = osteoarthritis.

*HA* = hemiarthroplasty.

*THA* = total hip arthroplasty.

*IM* = intramedullary.

In order to further evaluate factors possibly influencing the risk for reoperation Logistic regression analysis was performed. Age, gender, cognitive function, fracture type, fracture displacement, fracture reduction and implant position were first tested as independent variables in univariable models, and then a multivariable analysis adjusted for all the variables was performed. The only variables with a significantly increased risk for reoperation were displaced fracture preoperatively and poor position of the implant postoperatively (Table 3). Age, gender, cognitive function, fracture type or fracture reduction had no significant influence on the risk for reoperation.

**Table 3:** Logistic regression to evaluate risk factors for reoperation

	**No reoperation n = 113**^**a**^	**Reoperation n = 24**	**Multivaiable Logistic regression OR (95% CI) p-value**
**Age n (%)**			
−80	42 (75)	14 (25)	1^b^
81-	71 (88)	10 (12)	0.4 (0.2-1.2) 0.1
**Gender n (%)**			
Female	64 (83)	13 (17)	1^b^
Male	49 (82)	11 (18)	1.0 (0.4-2.9) 1.0
**Cognitive dysfunction n (%)**			
No	90 (81)	21 (19)	1 ^b^
Yes	23 (89)	1 (12)	0.4 (0.1-1.9) 0.3
**Primary fracture type n (%)**			
Basicervical	78 (84)	15 (16)	1^b^
Pauwels type 3	35 (80)	9 (21)	1.1 (0.4-3.2) 0.9
**Displaced fracture n (%)**			
No	32 (97)	1 (3)	1 ^b^
Yes	81 (78)	23 (22)	11 (1.4-86) 0.02
**Fracture reduction n (%)**			
Adequate	102 (85)	18 (15)	1 ^b^
Poor	14 (78)	4 (22)	0.6 (0.1-2.3) 0.4
**Implant position n (%)**			
Adequate	102 S(85)	18 (15)	1 ^b^
Poor	10 (63)	6 (38)	4.1 (1.1-15) 0.04

^a^ one patient died before postoperative radiographs were obtained.

^b^ reference.

*OR* = odds ratio for multivariable Logistic regression.

The most common remark on the implant position was that the SHS was positioned in the ventral area of the femoral head (n = 12), followed by penetration of the ARS (n = 2), cranial placement of the ARS in the femoral head (n = 1) or a distance between the tip of the screw and the bone/cartilage interface of >20 mm (n = 1).

When comparing the length of the SHS plate, no significant difference in reoperation rate was found comparing 2-hole and 4-hole plates; 19% (23/120) and 5.9% (1/17) respectively (p = 0.3).

The mean operative time was 65 (27-135) min, and the mean intra-operative blood loss was 183 (25–2300) ml.

The 6 month mortality was 25% (32/127), and the 1 year mortality was 31% (43/127). One patient died before postoperative radiographs were obtained.

## Discussion

Treatment of femoral neck fractures with a vertical orientation is a challenge, a fact that is highlighted by the high reoperation rate in this study – 18%. Although there are few recent studies, others have reported similar results of these rare fractures. In a study by Parker and Dynan [6] radiographs were analysed retrospectively in 151 patients with Pauwels type 3 fractures, and the incidence of non-union was found to be 29%. Saarenpää et al. [3] reported 7 reoperations in 30 patients (23%) with a basicervical fracture in a retrospective study. Furthermore, Liporace et al. [7] reported non-union or osteonecrosis in 16/42 (38%) patients suffering from a Pauwels type 3 fracture.

As a contrast, in a prospective cohort study by Massoud [5] on 41 patients with a basicervical (n = 13) or a trochanteric fracture (n = 28), only one screw penetration not needing surgery was reported, unfortunately not stating whether it was a basicervical or a trochanteric fracture patient.

### Fracture displacement

Displaced fractures had an increased risk for reoperation (22%) compared to undisplaced (3%) ones, and cut out or non-union was the most common reasons for reoperation among the patients with displaced fractures. There is no widely used classification system for displacement of basicervical fractures, most likely due to the controversies regarding whether they should be considered as intra- or extracapsular fractures [3]. However, our finding corresponds well to the results of Garden [4], and the Garden classification is well applicable also on intracapsular fractures with high Pauwels angle, ie type 3 fractures. The same finding was reported by Parker and Dynan [6] in their series of 151 Pauwels type 3 fractures where the displaced fractures had a non-union rate of 33% and the undisplaced 14%. Liporace et al. [7] reported that all undisplaced Pauwels type 3 fractures healed, although the number of patients was small (n = 4), in comparison to displaced fractures of which 28% (16/58) did not heal. With such a high risk for reoperation for the displaced fractures after internal fixation (22%), a primary arthroplasty might be an alternative [8].

### Reduction and implant position

We found a correlation between poor implant position and an increased risk for reoperation. This corresponds well with the literature on trochanteric fractures were the tip-apex distance [9] has been shown to be an important factor for predicting failure [10]. The most common error in our cohort was that the SHS was placed ventrally in the femoral head, which may increase the risk for cut out. This is an avoidable error and highlights the importance of proper use of intraoperative fluoroscopy for accurate placement of the screws.

We could not demonstrate an increased risk for reoperation in patients with poor fracture reduction. This is surprising and contrary to the findings of Liporace et al. [7]. They reported a non-union in 2/3 patients with fair or poor reduction compared to 8/59 patients with good or excellent reduction in patients with Pauwels type 3 fractures. One possible explanation could be that the classification of fracture reduction, although widely used, has poor validity and is unable to discriminate adequate from poor reduction. We still believe that adequate reduction of these fractures is of vital importance.

### Influence of the implant

Several principles and implants have been used to treat vertical femoral neck fractures over the years. In this study all patients have been operated upon using a combination of a sliding hip screw with a plate, which provides fixed angle stability to counteract the sheer force and varus displacement, and an additional antirotation screw.

There are few clinical studies that include only one implant, or perform sub-analyses of the influence of the implants if several implants are used. All 41 patients in a study by Massoud [5] were operated upon using the same method as in this study. Their results were exceptionally good; no reoperation, no mortality and no general complication after 12 months. This is in contrast to the radiographic study by Parker and Dynan [11] with 29% non-unions where all the 151 patients with Pauwels type 3 fractures were treated with 3 parallel screws. Liporace et al. [7] used several different types of implants (screws alone, different sliding hip screws, intramedullary nails) in Pauwels type 3 fractures, and reported that the failure rate was 19% for screws alone, and 8% for fixed angel devices.

In addition there are biomechanical studies that provide some support for the use of the present method. Blair et al. [2] created basicervical fractures in cadavers to compare screws alone, with SHS ± ARS. Their conclusion was that a SHS should be used, but they could not demonstrate any additional advantage from using an additional ARS. A similar result was reported by Aminian et al. [12] who studied simulated Pauwels type 3 fractures in cadavers, and found that a fixed angle device (locking femoral plate, dynamic condylar screw or SHS) provided a stronger construction compared to screws alone.

Most of the patients were operated upon using a 2-hole plate rather than a 4-hole plate. Although there was no statistical difference in the reoperation rate between the different plates, it can be assumed that a longer plate is more favorable from a biomechanical point of view, and the additional surgical trauma caused by a longer plate is most likely insignificant.

### Strengths and limitations

Strengths of this study are the large number of consecutively included patients treated with the same surgical method, the relatively long follow up time and the validation of the data using the National Board of Health and Welfare’s national registry. Limitations includes the retrospective design of the study, the lack of long term clinical and radiological follow-up and the lack of an independent blind observer for the radiological assessment. However, since our reoperation data are thoroughly validated we believe that our results provide relevant information regarding the treatment of these uncommon fractures

## Conclusions

Treatment of femoral neck fractures with a vertical orientation is problematic. The combination of a sliding hip screw and an antirotation screw was associated with a high reoperation rate – 18% for all, and 22% for displaced fractures. As a consequence, a primary arthroplasty might be the best option for patients with displaced fractures. Furthermore, accurate implant position was found to be of vital importance to avoid failure.

## Competing interests

The authors declare that they have no conflict of interest.

## Authors’ contributions

Both authors designed the study, analysed the data, wrote and finally approved the manuscript. LJL analysed all radiographs.

## Pre-publication history

The pre-publication history for this paper can be accessed here:

http://www.biomedcentral.com/1471-2474/13/171/prepub
